# Evaluation on the implementation effect of public participation in the decision-making of NIMBY facilities

**DOI:** 10.1371/journal.pone.0263842

**Published:** 2022-02-18

**Authors:** Hui Zhao, Yuanyuan Ge, Jingqi Zhang

**Affiliations:** School of Management Engineering, Qingdao University of Technology, Qingdao, China; Gonbad Kavous University, ISLAMIC REPUBLIC OF IRAN

## Abstract

The construction of NIMBY (Not in my backyard) facilities has caused many conflicts but is struggling to reduce it in China. With the background of public participation in social governance in the future, effective public participation is extremely helpful to solve this issue. Promoting public participation and scientifically evaluating the implementation effect of public participation are urgent problems to be solved at present. This study aims to analysis the factors hindering public participation and improve the implementation effect. Therefore, an evaluation system with 16 factors is established based on literature review and questionnaire survey, namely the basis of participation, participation process, external support, and cost-effectiveness. Interactions among the 16 factors are further evaluated by expert opinions. The objective and subjective weights of indicators are determined and combined by introducing Decision-Making Trial and Evaluation Laboratory (DEMATEL) and entropy weight method (EWM). Considering the uncertainty and randomness of subjective judgment, cloud model is introduced to evaluate the implementation effect of public participation. Finally, this framework is applied to evaluate the project of Jiu Feng waste-to-energy (WTE) plant in Hangzhou, China, which verifies the applicability of the evaluation framework for the implementation effect of public participation in NIMBY facilities. The results indicate that the implementation of public participation is between "average" and "good", with significant room for improvement in the involvement of NGOs and the influence of public opinion on decision-making. Additionally, the participation process has a significant impact on the whole system. The framework can provide government departments with guidance in implementing public participation.

## 1. Introduction

The process of industrialization and urbanization is advancing rapidly in the world. In China, with the comprehensive deepening of reform, the urbanization level has increased from 36.09% (2000) to 63.89% (2021) and is expected to reach 70.99% in 2030 [1]. The accelerated urbanization has brought a massive increase in the urban population. To meet the growing needs of the citizens for production and life, the government has invested heavily in the construction of production facilities, service facilities, and infrastructure. More and more large-scale public projects have begun construction, such as sewage treatment plants, garbage treatment plants, and power plants, which are beneficial to most people. However, these projects have negative impacts on the residents around the construction site, and are often not welcomed by the surrounding residents. Such projects with obvious negative external effects are defined as NIMBY facilities [2]. As the most common NIMBY facilities with the highest NIMBY effect, polluting NIMBY facilities are more likely to trigger public sensitivity and resistance due to their greater potential danger and pollution. At the same time, people’s democratic consciousness and cognitive level gradually increase, they believe that the construction and operation of these facilities produce environmental pollution, which may adversely affect the living environment and health. Therefore, NIMBY conflicts are increasing yearly, no matter in developed or developing countries, collectivism institution or democracy institution [3–5]. For instance, more than a decade of conflict between Hazardous waste management and public resistance in Portugal [4], nearby residents took to the streets in revolt due to building the Hubei Yangluo WTE plant in 2019. Regarding the resolution of the NIMBY conflicts, Ibitayo believed that public participation is a prerequisite in the construction of NIMBY facilities [6]. Public trust and early, continuous participation in the facility siting process can increase the likelihood of successful siting [7]. Yong Liu [8] also pointed out that public participation is an effective means for the government to improve the public acceptance of waste incineration facilities by residents. And the government should provide more opportunities for the public to participate in decision-making [9, 10]. Studies above have shown that the necessity of public participation has been universally recognized [11], and public participation can reduce conflicts caused by NIMBY and protect public interests. Moreover, public participation can improve decision ability of decision-makers [12], then to make them know the public’s concerns [13].

Public participation is essential to solving NIMBY conflicts [11]. Measuring the implementation effect of public participation and reaching high effectiveness in public participation has consistently drawn the attention of researchers. Sun et.al [14] took Shanghai and Hong Kong as examples, adopted qualitative methods to measure the implementation effect of public participation, and suggested that key stakeholders should be involved in improving the level of public participation. Rowe and Frewer [15] established a framework to evaluate the effectiveness of public participation methods, mainly from the acceptance criteria and process criteria, but the research remains at the level of qualitative discussion. Some scholars [10, 16] also formulated a zoning framework for discourse power during the site selection, construction, operation, and final abandonment of NIMBY facilities to maximize the participation of public groups. Unfortunately, most scholars focus on promoting public participation. They fail to quantitatively measure the implementation effect of public participation, which leads to lack of persuasiveness. There are several outstanding problems. Firstly, although research results are relatively abundant in identifying the influencing factors of public participation, the research on the evaluation index system of the implementation effect of public participation in the decision-making of NIMBY facilities is limited. Secondly, characteristics of NIMBY facilities vary with different types [17], and few researches focus on polluting NIMBY facilities, which conflicts account for more than 60% in China. Therefore, it is necessary to select polluting NIMBY facilities as case study. Finally, most researches adopt the qualitative analysis method in the evaluation, but few quantitative studies are available. The qualitative analysis has intense subjectivity and poor assessment findings. Consequently, it is still a worthy research topic about the implementation effect of public participation and what hinders public participation in the decision-making process of polluting NIMBY facilities. This research can fill the gap.

**Fig 1:**
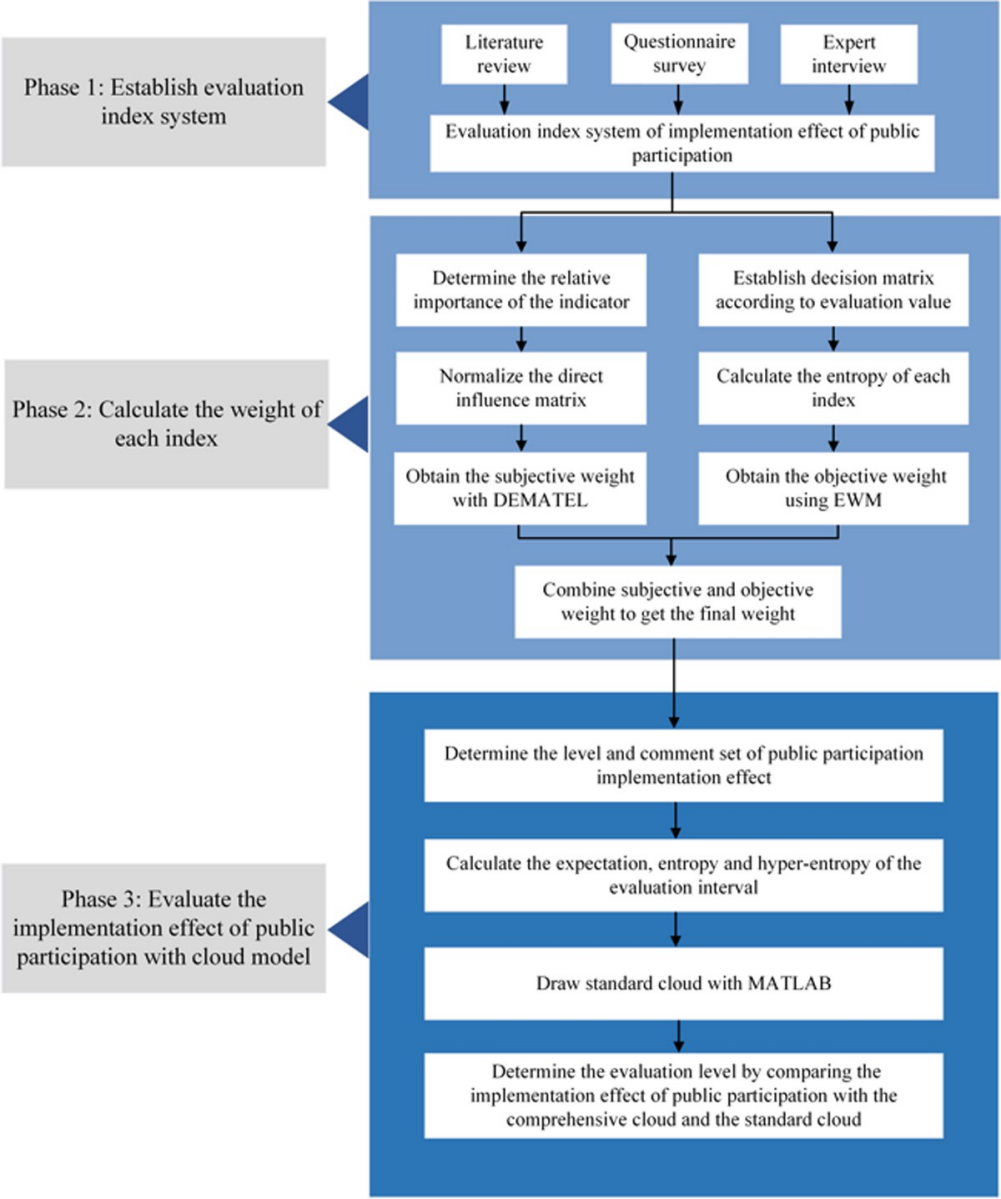
The evaluation framework of the implementation effect of public participation.

To evaluate the implementation effect, and improve the effectiveness of public participation, which can help the government find the critical factors affecting public participation, evaluation framework constructed as shown in Fig 1. This paper makes several innovative and contributive endeavors as follows. (1) Formulate a comprehensive indicator system. Factors hindering the promotion of public participation are screened from existing literature. Then combining questionnaires and expert views, this paper systematically identifies the factors affecting public participation in China. (2) NIMBY is a wicked problem.

Considering the correlation of influencing factors, the DEMATEL method is used to analyze the causal relationship,which is subjectively method. Therefore, the EWM is added to correct the weights. (3) Propose a new approach to quantitatively evaluate the implementation effect. Cloud model is introduced to improve the accuracy due to fuzziness and hesitation of public judgement, which tolerates inevitable and arbitrary decisions. This systematic study identifies the barriers affecting the implementation effect of public participation and provides policymakers with references to optimize the public participation system.

The remaining section is as follows. The second section introduces the NIMBY facilities and comprehensively combs the research status of public participation in the decision-making of NIMBY facilities. In the third section, we construct an evaluation index system. The fourth section analyzes the implementation effect of public participation based on combination weighting and the cloud model. The fifth section verifies the feasibility and effectiveness of the proposed evaluation framework through practical case analysis. In the sixth section, sensitivity and comparative analysis are carried out. Finally, conclusions and further work are given.

## 2. Literature review

### 2.1. NIMBY facilities

The concept of “NIMBY facilities” was first proposed by O’Hare to describe those facilities that can bring benefits to the whole society but have negative impacts on the surrounding residents [17]. In 1980, the British journalist Livezey first introduced the theory of NIMBY, which meant “Not in my backyard”, which was then widely used [4]. China’s research on NIMBY facilities started relatively late and was initially introduced into the mainland by Taiwan scholars. Since then, many scholars in China have carried out a localized definition. Li interpreted it as a project that can serve most people, but because it may threaten health, living environment, life, and property so that residents do not want to build projects near their homes [18]. He recognized that NIMBY facilities are usually referred to as some facilities with pollution threats, such as thermal power plants and landfills, etc [2]. Yang [19] believed that NIMBY facilities refer to facilities unwilling acceptable locally, but are indispensable for realizing social public welfare. The classification of NIMBY facilities by scholars is different from different perspectives. As shown in Table 1, several categories are summarized.

**Table 1:** NIMBY facilities classification.

Scholars	Classification of Angle	Type	Description
O’Hare [17]	Cost to benefit ratio	Hopeless of success	Facilities can be broken for almost anyone
		Turkey	Facilities do great harm to neighbors near the site of construction
		Unfair	Facilities do more harm to residents than they benefit
		Classic NIMBY	The costs of the facilities are imposed on the residents, while the benefits are shared by the community
		Free lunch	Facilities are beneficial to the entire community
Li Y, He J [18]	NIMBY effect	No NIMBY effect	Such as city parks, libraries and so on
		Mild NIMBY effect	Such as cultural and educational facilities, schools, stations, and so on
		Moderate NIMBY effect	Such as nursing homes, STD prevention and treatment centers, highways, markets, etc
		High NIMBY effect	Such as garbage incinerators, sewage treatment plants, petrochemical plants, gas stations, etc
Tao P, Tong X [20]	Loss of dimension	Pollution of the class	Such as waste incineration sites, magnetic levitation, airports
		Risk cluster class	Such as nuclear power plants, chemical plants, gas stations
		Stigmatized class	Such as drug rehabilitation centers, prisons, infectious disease hospitals
		Psychological unhappiness	Such as a funeral home or crematorium or cemetery

The above study found that the characteristics of different types of NIMBY facilities are significantly different, which has a noticeable impact on the enthusiasm of public participation. Public participation is required for the smooth construction of polluting NIMBY facilities. With the current rapidly developing circumstances in China, polluting NIMBY facilities need extensive construction. Therefore, this paper selects polluting NIMBY facilities as a case study and defines them as facilities that are potentially dangerous and polluting to the surrounding environment and produce air, water, soil, and noise pollution during their construction or operation, such as waste-to-energy plants, garbage treatment plants, sewage treatment plants, chemical plants, etc.

### 2.2. Public participation in the decision-making of NIMBY facilities

Public participation in the decision-making stage of NIMBY projects can effectively prevent NIMBY conflicts [8]. In order to enable the public to better participate in the decision-making of NIMBY facilities, many scholars have explored the key reasons, and some believe that the government level is one of the critical reasons. The government thinks the introduction of public participation inevitably affects the achievement of the economic goals [21], and fails to pay attention to the feedback from the public, which eventually leads to the forced relocation of the project [22, 23]. Yu et.al [24] suggested changing the previous public participation model, from the traditional government-led model to the public-led model, which can convey the public’s interests to decision-makers. As the initiator and supervisor of the construction of NIMBY facilities, government departments should establish a tripartite communication channel among nearby residents, construction companies, and the government, which can effectively promote the development of NIMBY facilities [8].

In addition, some scholars have studied the factors affecting public participation from the perspective of the participation process. Soma and Yao et.al [25, 26] pointed out that the selection of public participation representatives should be more extensive, including not only the social elite but also the general public. The limitation of public representatives cannot represent the general public, ultimately leading to ineffective participation [12]. Participation skills [27] and procedural processes directly impact the effectiveness of public participation. Furthermore, whether the relevant information is open and transparent in the decision-making process of NIMBY facilities also has a significant impact on public participation [28]. To fundamentally improve public participation at the present stage is to enhance their awareness of environmental protection and focus on carrying out environmental protection education [29]. Participation mechanism, technology choice, and economic factors are necessary to enhance the willingness to participate [30]. Online participation methods can also be added, such as online questionnaires, public symposiums, and debates through video conferencing [31]. While reducing the cost of participation, online participation methods enable the public to express their opinions more truthfully. In conclusion, public participation can improve the scientific nature of environmental planning, make it more accurate to reality, improve the implementation ability of the plan. Accordingly, strengthening legislation [14] and establishing engagement guidelines [32], providing multiway interaction and training, and conducting education programs are all necessary to enhance the effectiveness of public participation.

Until now, several scholars have studied the evaluation of the implementation effect of public participation, as shown in Table 2. These studies usually qualitatively consider different procedural aspects as observable variables, such as the timing and duration of the participation process, the provision of information to the public, the arrangement of public consultation, or quantitatively measure the implementation effect of public participation through an index. The factors mentioned above have contributed to improving public participation. However, they do not consider the interplay of variables and the difference in weights. Most importantly, they do not directly involve the public in evaluating the implementation effect. There is still s lack of comprehensive analytical framework that integrates the different influencing factors and incorporates the perspectives of experts and the local public.

**Table 2:** Research on the implementation effect of public participation.

Scholars	Evaluation criteria	Case study	Qualitative or quantitative methods
Sang [33]	Categories of the public participation, the power given to the public, time of public participation, methods of conflict resolution	China	Qualitative
Rowe and Frewer [15]	Acceptance criteria (representativeness of participants, independence of true participants, early involvement?, influence on final policy, transparency of process to the public), Process criteria (resource accessibility, task definition, structured decision making, cost-effectiveness)	the UK	Qualitative
Nadeem and Fischer [34]	Legal requirements, information, timing and venue of public consultation, composition and awareness of the public involved, methods of consultation, incorporation of public concerns into the final decision, and transparency of decision making process	Pakistan	Qualitative
Mwenda et.al [35]	Notifification processes, participation methods, venue of participation, language used, type of participants	Kenya	Quantitative
Daniele and Angela [32]	Timing, information provision, consultation arrangements, public consulted, incorporation of consultation results in EIA report	China	Quantitative
Wu et.al [36]	The social responsibility of stakeholders, the behaviors of stakeholders	China	Quantitative

By summarizing the existing literature, it can be found that scholars have conducted extensive research on promoting participation. Most of them have focused on identifying the key factors affecting public participation and achieved specific results but have not further explored the internal relationships among the factors. A systematic framework for evaluating the implementation effect of public participation has yet to be formed. Therefore, it is essential to quantitatively measure the implementation effect of public participation, analyze the influencing factors that hinder public participation, and present the current problems. The results can provide local governments with ideas and guidance to reduce NIMBY conflicts.

## 3. Establish an evaluation index system

In this section, the factors influencing the implementation effect of public participation are extracted through literature review, expert consultation, and questionnaires. Several scholars have researched how to evaluate the implementation effect of public participation (Table 2). Rowe et.al [15] divided the factors into acceptance criteria, which concern whether the implementation of public participation is as effective as it should be, and process criteria, which concern the reasonableness of the participation process. Nadeem et.al [34] divided the influencing factors into four categories: environment, method, impact, and content, their study targeted developing countries, and the researchers also subdivided the factors in each category. In addition, the International Association for Public Participation proposed core values of public participation to facilitate the process and implementation of public participation, while listing several essential indicators that determine the success of public participation (Table 3).

**Table 3:** Indicators in IAP2 research.

No.	Indicators	Explain
1	Attitude	Allow the public to participate directly in projects that affect their own interests
2	Empowerment	Give the public the right to participate, but the public should use it reasonably
3	Appeal	Interest demands of various stakeholders
4	Identify participants	Identify all stakeholders
5	Participation stage	Identify stages of public participation
6	Provide information	Provide information serviceable to participants through appropriate channels
7	Feedback	Feed back the opinions of participants and then affect decision-making

After sorting the literature and other materials, 18 influencing indicators of the implementation effect of public participation were initially screened, listed in S1 File.

Based on S1 File, the factors influencing the effect of public participation were modified by consulting the public, governmental staff, and experts from various disciplines such as construction, energy, environment, and politics. The factors of “social and economic status of the public” and “public’s understanding of NIMBY facilities” were removed, as they could be summarized by “public awareness of participation”. Finally, 16 influencing factors were determined, and the questionnaire was designed using the above influencing factors (S2 File). A total of 240 questionnaires were distributed, 223 were recovered, and 218 were valid after processing, with an effective recovery rate of 90.83%. After collecting and sorting out the questionnaire data, the basic descriptive statistics obtained are shown in Table 4. The questionnaires were filled by relevant government departments, NIMBY facility construction enterprises, the public, environmental assessment departments and Non-Governmental organizations. Their working years ranged from less than five years to more than 15 years, with broad coverage and reliable results. The information of the respondents is presented in Table 5.

**Table 4:** Descriptive statistics.

Factors	N	Min	Max	Mean	Standard deviation
Project information openness and transparency	218	1	5	3.560	0.718
The government’s attitude towards public participation	218	1	5	3.706	0.716
The soundness of relevant laws and regulations	218	1	5	3.449	0.737
A mechanism for receiving public feedback	218	1	5	3.422	0.626
The level of attention from the news media	218	1	5	3.660	0.654
Convenience of project information access	218	1	5	3.018	0.650
Public awareness of participation	218	2	5	3.904	0.817
The interactivity of public participation	218	1	5	3.404	0.660
Representation of the main body of public participation	218	1	5	3.876	0.754
Continuity of public participation	218	1	5	2.917	0.624
Transparency in the participation process	218	1	5	3.431	0.649
Involvement of NGOs	218	1	5	3.101	0.628
The role of relevant experts	218	1	5	3.128	0.687
The objective attitude of EIA agencies	218	1	5	2.963	0.698
Cost consumption in public participation	218	1	5	3.638	0.700
The influence of public opinion on decision-making	218	1	5	3.748	0.676

**Table 5:** Demographic characteristics of respondents.

Characteristics	Number	Percentage
Gender		
Male	147	67.43%
Female	71	32.57%
Age		
20–29years	64	29.36%
30–39years	105	48.17%
40–49years	36	16.51%
≥50years	13	5.96%
Work experience		
0–5 years	50	22.94%
6–10 years	116	53.21%
≥11 years	52	23.85%
Educational background		
High school and below	17	7.80%
Associate college	32	14.68%
Bachelor’s degree	129	59.17%
Master’s degree and above	40	18.35%

In order to establish the index system for evaluating the implementation effect of public participation in the decision-making of NIMBY facilities, this paper uses factor analysis to reduce the dimensionality of the influencing factors. Factor analysis is a multivariate statistical analysis method that describes multiple observable and related variables with a small number of non-observed obtained factors, and we apply SPSS 22 for factor analysis. After testing, Cronbach’s Alpha = 0.713, KMO = 0.736, the approximate chi-square value of Bartlett’s sphere test is 879.264, Sig = 0, indicating that the sample data in this study is suitable for factor analysis. To extract common factors and classify indicators, this paper uses principal component analysis to analyze the covariance matrix of independent variables. In this process, the Kaiser-Rule-Thumb was widely used, and this method takes the components with eigenvalues greater than 1 as principal components, because the contribution of factors with eigenvalues less than 1 may be small. According to the degree of variance explanation, four common factors with eigenvalues greater than 1 are finally selected. As shown in Table 6, the degree of variance interpretation is 56.567% > 50%, which shows that the selected four main factors can represent more than half of the information of the original evaluation index. Therefore, they can be used as the main factors affecting the implementation effect of public participation.

**Table 6:** Description of total variance.

Component	Initial eigenvalues			Rotation sums of squared loadings		
	Total	Variance (%)	Cumulative (%)	Total	Variance (%)	Cumulative (%)
1	3.322	20.765	20.765	2.849	17.807	17.807
2	2.461	15.382	36.147	2.430	15.189	32.996
3	1.916	11.973	48.120	1.938	12.115	45.111
4	1.352	8.447	56.567	1.833	11.456	56.567

By setting up the initial factor loading matrix, combined with the maximum variance rotation method, the structure is optimized and integrated to facilitate factor interpretation and highlight the typical representativeness of each common factor. In Table 7, factor 1 includes project information openness and transparency, the government’s attitude towards public participation, the soundness of relevant laws and regulations, a mechanism of receiving public feedback, the level of attention from the news media and the convenience of project information access, which are the most basic conditions for public participation, and therefore named as the basis of participation. Factor 2 includes public awareness of participation, the interactivity of public participation, representation of the main body of public participation, continuity of public participation, and transparency in the participation process, which are all related to the process of public participation and therefore named as the participation process. Factor 3 covers the involvement of NGOs, the role of relevant experts, and the objective attitude of EIA institutions. To some extent, experts and NGOs are not exactly the direct participation subjects, but provide help in knowledge, technology, and theory from the perspective of a third party, so this category of factors is named external support. Cost consumption in public participation and the influence of public opinion on decision-making are included in factor 4, which represents the cost consumed and the effect achieved in public participation respectively, so named as cost-effectiveness. Accordingly, the evaluation system is obtained (Table 8).

**Table 7:** The rotated component matrix.

Factors	Factor analysis			
	1	2	3	4
Project information openness and transparency	.783	.005	.039	-.115
The government’s attitude towards public participation	.649	.171	-.172	.068
The soundness of relevant laws and regulations	.779	-.149	.092	-.033
A mechanism for receiving public feedback	.615	-.080	.066	.367
The level of attention from the news media	.609	.041	-.093	.446
The convenience of project information access	.623	.037	.157	.215
Public awareness of participation	-.009	.667	-.076	.004
The interactivity of public participation	-.028	.658	.096	.253
Representation of the main body of public participation	-.017	.645	.095	.250
Continuity of public participation	.150	.590	.028	-.298
Transparency in the participation process	-.045	.803	-.064	.044
The involvement of NGOs	.061	.022	.716	-.074
The role of relevant experts	.006	-.094	.763	.176
The objective attitude of EIA agencies	.009	.108	.856	-.010
Cost consumption in public participation	.136	.036	.022	.804
The influence of public opinion on decision-making	.156	.243	.055	.730

**Table 8:** Evaluation index system of the implementation effect of public participation.

First-level factors	Second-level factors	Index description
B_1_: The basis of participation	C_1_: The soundness of relevant laws and regulations	Refers to the adequacy of laws and regulations that specifically address public participation in NIMBY facilities [32]
	C_2_: Project information openness and transparency	Refers to whether all information relating to the construction of the NIMBY facility is disclosed in a timely manner [9]
	C_3_: The government’s attitude towards public participation	Refers to whether the government organizes professional training or consultation meetings for the public and whether it has a active attitude towards public participation [36]
	C_4_: A mechanism for receiving public feedback	Refers to the ability of the government to accept and respond to public opinion [32]
	C_5_: The level of attention from the news media	Refers to whether the media is monitoring the reporting of critical events and ensuring the authenticity of the reporting [37]
	C_6_: The convenience of project information access	Refers to whether the information disclosed by the NIMBY facility construction enterprise and the government is easy to obtain [38]
B_2_: Participation process	C_7_: Public awareness of participation	Refers to the degree of public awareness and concern about NIMBY facilities, and whether the public has the intention to participate in the construction of NIMBY facilities [25]
	C_8_: The interactivity of public participation	Refers to whether the public and the government can carry out information interaction [32]
	C_9_: Representation of the main body of public participation	Refers to the fact that the public participants are representative enough to express the opinions of the public [35]
	C_10_: Continuity of public participation	Refers to whether the public has time to continue to participate in the decision-making of NIMBY facilities [15]
	C_11_: Transparency in the participation process	Refers to whether the process of public participation is open and transparent [3]
B_3_: External support	C_12_:The involvement of NGOs	Refers to the ability of environmental NGOs to act as a bridge between the government and the public while remaining neutral [34]
	C_13_: The role of relevant experts	Refers to the objectivity and impartiality of experts involved in the decision-making of NIMBY facilities [37]
	C_14_: The objective attitude of EIA agencies	Refers to the neutrality of the environmental assessment agency during the review process [39]
B_4_: Cost-effectiveness	C_15_: Cost consumption in public participation	Refers to the consumption of time and money for the participation [40]
	C_16_: The influence of public opinion on decision-making	Refers to whether public opinion is taken into account in the final decision [34]

## 4. Research methodology

### 4.1. Combination weight method is adopted

The weights of influencing factors in the evaluation are different. To achieve a scientific evaluation of the implementation effect of public participation, it is particularly significant to assign weight to the index reasonably. The determination of weight directly affects the accuracy of the evaluation results. The current research mainly includes subjective and objective weight, but their mechanisms are disparate, and the evaluation results also have their advantages and disadvantages. Subjective weight relies too much on the opinions of experts and is affected by personal knowledge, while objective weight only focuses on mathematical-statistical methods and ignores the influence of people. Therefore, to highlight the critical role of expert experience and consider the objective information reflected by the index data, this research combines the two methods. For the analysis of public participation implementation effect, DEMATEL is an enormously advantageous method, which can consider the influence of factors synthetically. In order to calculate indicator weights more scientific and rational, EWM, which is an objective weighting method, is introduced to correct the DEMATEL results.

#### 4.1.1. DEMATEL method

The DEMATEL method was developed by the U.S. National Laboratory from 1972 to 1976. It mainly solves complex social problems in real life by comprehensively combining the knowledge and experience of experts in related fields [41]. This method employs matrix tools and graph combination methods to analyze the interaction degree of indicators in the evaluation index system [42]. By analyzing the logical relationship and influence matrix between the indicators in the evaluation system, the influence degree between each indicator and other indicators can be obtained. Furthermore, we also acquire the centrality and cause degree of each indicator [43]. The steps to determine subjective weights based on the DEMATEL method are as follows.

Step 1: Initialize the direct impact matrix *A*. Experts are invited to score the degree of mutual influence among evaluation indicators, where *d*_*ij*_ represents the degree of direct influence of index *X*_*i*_ on index *X*_*j*_. Arithmetical average processing is carried out on all expert scores to form the initial direct impact matrix *D*

$$D=\left[\begin{array}{cccc} d_{11} & d_{12} & \cdots & d_{1n}\\ d_{21} & d_{22} & \cdots & d_{2n}\\ \ddots & \ddots & \ddots & \ddots \\ d_{n1} & d_{n2} & \cdots & d_{nn} \end{array}\right]$$ (1)

Step 2: Normalize the direct impact matrix. The direct impact matrix is normalized via Eq (2) to obtain the normalized direct impact matrix *S*

$$S={{(s}_{ij})}_{n\times n}={D(\underset{0\leq i\leq n}{\max}\sum_{j=1}^{n}d_{ij})}^{-1}$$ (2)

Step 3: Calculate the comprehensive impact matrix *T* among the evaluation indicators

$$T={{(t}_{ij})}_{n\times n}={S(1-S)}^{-1}$$ (3)

Step 4: Calculate the effect degree *E*_*i*_, affected degree *P*_*i*_, center degree *M*_*i*_, and cause degree *C*_*i*_. The effect degree represents the influence of factor *X*_*i*_ on all others factors, its value $E_{i}=\sum_{i=1}^{n}T_{ij}(i=1,2,3\ldots n)$; the affected degree indicates the comprehensive influence degree of factor *X*_*i*_ by all other factors, its value $P_{i}=\sum_{j=1}^{n}T_{ij}(j=1,2,3\ldots n)$; the center degree reflects the position of this factor among all factors, and its value indicates the effect degree of this factor. The larger the value, the more obvious the influence, and vice versa, its calculation formula is: *M*_*i*_ = *E*_*i*_+*P*_*i*_(i = 1,2,3…n); the cause degree is the effect degree minus the affected degree, and its value can be positive or negative. If it is negative, this factor is the result factor, which means it is more affected by other factors; if it is positive, then this factor is the cause factor, which means it has more influence on other factors, and its calculation formula is as follows: *C*_*i*_ = *E*_*i*_−*P*_*i*_(i = 1,2,3…n).

Step 5: Determine the subjective weight of each indicator $w_{k}^{\prime }$

$$w_{k}^{\prime }=\sqrt{{M_{i}}^{2}+{C_{i}}^{2}}/\sum_{i=1}^{n}\sqrt{{M_{i}}^{2}+{C_{i}}^{2}}$$ (4)

#### 4.1.2. Entropy weight method

In information theory, entropy measures the degree of disorder in the system, which can estimate the amount of effective information provided by data [44]. EWM is a mathematical method that comprehensively analyzes the information provided by each indicator to determine the weight. The smaller the entropy value, the greater the degree of dispersion of the indicator, indicating that the indicator has a greater impact on the comprehensive evaluation. EWM can weaken the subjectivity of expert judgment to some extent [45, 46]. The steps to determine objective weights are shown below.

(1) Construct the decision matrix *B* = (*b*_*lk*_)_*m*×*n*_ to represent the evaluation index set, which indicates the weight evaluation value of the k-th index by the l-th expert, where *l* = 1, 2,…,m, *k* = 1, 2,…,n, where m and n represent the number of experts and the number of each evaluation index respectively.

(2) Calculate the entropy value. First, compute the proportion of each index value *r*_*lk*_, $\;r_{lk}=b_{lk}/\sum_{l=1}^{m}b_{lk}$, then compute the entropy of evaluation index respectively *H*_*k*_, which if *r*_*lk*_ = 0, regulation *r*_*lk*_*ln r*_*lk*_ = 0, have 0≤*H*_*K*_≤1.


$$H_{K}=\frac{-1}{\ln\;m}\sum_{l=1}^{m}r_{lk}\;\ln\;r_{lk}$$ (5)

(3) Since the evaluation value in this paper does not involve dimensions, the weight can be directly calculated by using the evaluation value. Then the weight of the k-th evaluation index $w_{k}^{\prime \prime }\;$ can be expressed as

$$w_{k}^{\prime \prime }=1-H_{K}/\sum_{K=1}^{n}\left(1-H_{k}\right)$$ (6)

#### 4.1.3. Determine the combination weight based on game theory

After calculating the weight by the DEMATEL and EWM, the idea of game theory is introduced to determine the combined weight finally. According to the thought of game theory, if the subjective weight of the index *w*′ = (*w*′_1_, *w*′_2_,…,*w*′_*k*_) and the objective weight *w*′′ = (*w*′′_1_, *w*′′_2_…,*w*′′_*k*_) is regarded as two sides of the game, and the optimal combined weight can be treated as the two sides of the game reach the equilibrium state [47]. From a mathematical perspective, in the balanced state, the sum of the deviation between *w*′ and *w*′′ and the combined weight of the index should be minimized. The procedures are as follows:

(1) Denote the index combined weight vector expressed by the linear combination of *w*′ and *w*′′ as

$$w=\left(\begin{array}{c} \lambda_{1}w_{1}^{\prime }+\lambda_{2}w_{1}^{\prime \prime }\\ \lambda_{1}w_{2}^{\prime }+\lambda_{2}w_{2}^{\prime \prime }\\ \vdots \\ \lambda_{1}w_{k}^{\prime }+\lambda_{2}w_{k}^{\prime \prime } \end{array}\right)=\lambda_{1}w^{\prime }+\lambda_{2}w^{\prime \prime }$$ (7)

Where *λ*_1_ and *λ*_2_ are linear combination coefficients.

(2) According to game theory, the objective function is established to minimize the sum of deviation between combination weight and *w*′ as well as *w*′′. Then seek the optimal linear coefficients $\lambda_{1}^{*}$ and $\lambda_{2}^{*}$. At this time, the index combined weight is the optimal weight w*. The objective function satisfies the following conditions:

$$\left\{ \begin{array}{c} \min\;({\left\|w-w^{\prime }\right\|}_{2}+{\left\|w-w^{\prime \prime }\right\|}_{2})=min\;({\left\|\lambda_{1}w^{\prime }+\lambda_{2}w^{\prime \prime }-w^{\prime }\right\|}_{2}+{\left\|\lambda_{1}w^{\prime }+\lambda_{2}w^{\prime \prime }-w^{\prime \prime }\right\|}_{2})\\ s.t.\;\lambda_{1}+\lambda_{2}=1\;\lambda_{1},\lambda_{2}\geq 1\; \end{array}\right.$$ (8)

(3) According to the differential theory, the conditions for the first derivative of Model (8) to obtain the minimum value are as follows:

$$\left\{ \begin{array}{c} \lambda_{1}w^{\prime }w^{\prime T}+\lambda_{2}w^{\prime }w^{\prime \prime T}=w^{\prime }w^{\prime T}\\ \lambda_{1}w^{\prime \prime }w^{\prime T}+\lambda_{2}w^{\prime \prime }w^{\prime \prime T}=w^{\prime \prime }w^{\prime \prime T} \end{array}\right.$$ (9)

(4) The linear combination coefficients $\lambda_{1}^{*}$ and $\lambda_{2}^{*}$ obtained by normalization processing:

$$\left\{ \begin{array}{c} \lambda_{1}^{*}=\frac{\left|\lambda_{1}\right|}{\left|\lambda_{1}+\lambda_{2}\right|}\\ \lambda_{2}^{*}=\frac{\left|\lambda_{2}\right|}{\left|\lambda_{1}+\lambda_{2}\right|} \end{array}\right.$$ (10)

So, we end up with $w^{*}=\lambda_{1}^{*}w^{\prime }+\lambda_{2}^{*}w^{\prime \prime }$.

### 4.2. Cloud model

Based on probability theory and fuzzy mathematics, Academician Li Deyi innovatively proposed a cloud model theory to deal with uncertain decision-making problems [48]. It uses membership cloud, digital features, cloud generators, and other means to realize the uncertainty conversion between qualitative language values and quantitative representations, effectively deal with the problems of randomness and ambiguity, and solve the shortcomings of traditional evaluation methods in this respect [49, 50]. At the same time, compared with the traditional evaluation methods, it is more consistent with objective facts. Li et al [51] through comparative research of cloud model, fuzzy comprehensive evaluation method, and BP neural network model, showed that the evaluation results obtained by cloud model are more accurate. In recent years, the cloud model evaluation method has been applied to the field of management. Some scholars have introduced the cloud model into the evaluation of investment. For example, Liu et.al [52] built a new energy investment assessment framework based on the cloud model. In addition, some scholars used the cloud model to evaluate risk. For instance, Yu et.al [53] evaluated the risk of submarine pipeline, using the VIKOR method is extended with the cloud model to determine the risk priority of failure modes. Abundant research results provide a certain reference for this paper. The evaluation of the implementation effect of public participation in the decision-making of NIMBY facilities is a complex problem, influenced by many factors, with uncertainty, fuzziness and randomness, and the cloud model can manage this problem well.

#### 4.2.1. Model principle

In the cloud model theory, the quantitative domain can generally be represented by *U*, then *C* is a certain qualitative expression of this domain *U*, a random occurrence on the qualitative expression *C* is represented by *x*, and the quantitative value *x*∈*U*. The degree of certainty of *x* to *C*, *μ*(*x*)∈[0,1], is a random number that tends to be stable.

Therefore, the cloud is used to represent the layout of *x* on the domain *U*, which is recorded as *C*(*x*). And x is a cloud drop on the domain *U*.

(1) Digital characteristics of cloud

The digital characteristics of the cloud model reflect the quantitative characteristics of qualitative concepts. The cloud map composed of cloud droplets in the cloud model is represented by three numerical values, denoted as *C*(*Ex*, *En*, *He*). Expectation *Ex* is the most representative point of qualitative concept in the domain space. It is reflected on the cloud map as the “highest point” of the cloud, that is, the point whose membership degree is 1. Entropy *En* is used to measure the degree of uncertainty of a qualitative concept, which is jointly determined by the randomness and ambiguity of the concept. The larger the entropy, the stronger the vagueness of the concept, and the more difficult it is to quantify it. The reflection on the cloud map indicates the “span” of the cloud, that is, the greater the entropy, the larger the “span” of the cloud. Hyper-entropy *He* represents the uncertainty of entropy, and it also indicates the randomness of the sample, namely the dispersion degree of cloud droplets on the cloud map. *He* correlates ambiguity and randomness, which is reflected on the cloud map to represent the "thickness" of the cloud. The greater the super entropy, the thicker the cloud.

(2) Cloud generator

**Fig 2:**
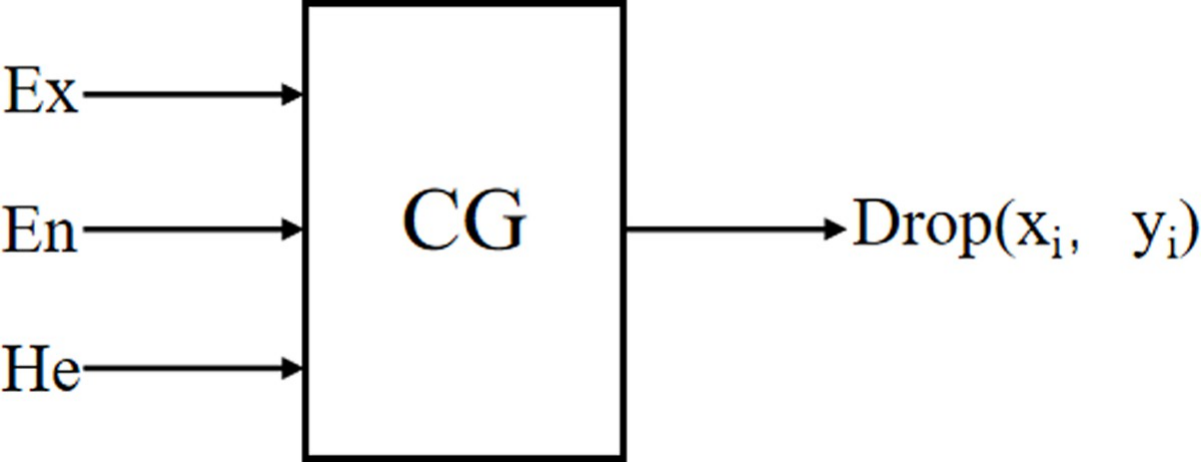
The forward cloud generator.

**Fig 3:**
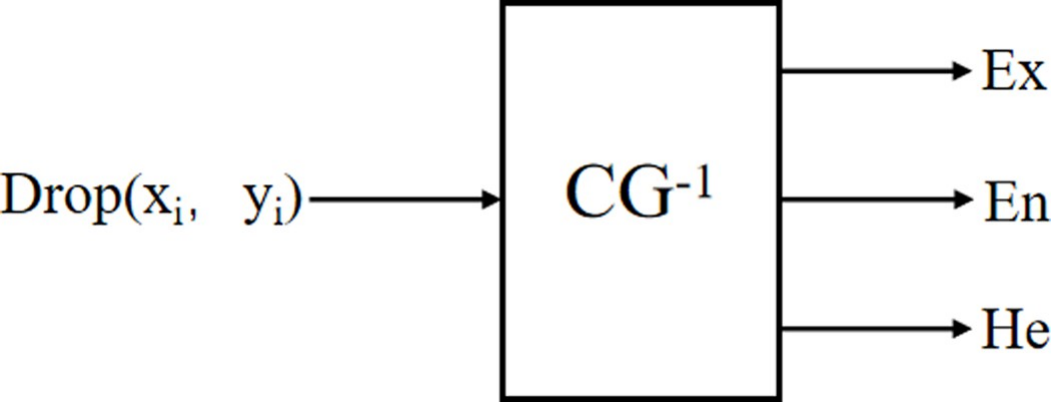
The reverse cloud generator.

The generator is a specific algorithm for the mutual conversion between qualitative concepts and quantitative data in the cloud model. The forward cloud generator realizes the conversion from qualitative concepts to quantitative values, that is, (*Ex*, *En*, *He*) is transformed into qualified cloud droplets, and the principle is shown in Fig 2. The reverse cloud generator realizes the transition of quantitative values to qualitative concepts, and the precise numerical input can be effectively transformed into (*Ex*, *En*, *He*), as shown in Fig 3. The algorithm of one-dimensional reverse cloud generator can be performed as follows:

Calculate the sample mean, where *N* is the number of cloud droplets generated in the generator

$$\bar{X}=\frac{1}{N}\sum_{i=1}^{N}x_{i}$$ (11)

$$Calculate\;the\;first‐order\;sample\;center\;distance:\frac{1}{N}\sum_{i=1}^{n}\left|x_{i}-\bar{X}\right|$$ (12)

$$(3)\;Calculate\;the\;sample\;variance\;S^{2}=\frac{1}{N-1}\sum_{i=1}^{N}{\left(x_{i}-\bar{X}\right)}^{2}$$ (13)

$$(4)\;Compute\;the\;eigenvalue:\;\left\{ \begin{array}{c} E_{x}=\bar{X}\\ E_{n}=\sqrt{\frac{\pi }{2}}\cdot \frac{1}{N}\sum_{i=1}^{n}\left|x_{i}-E_{x}\right|\\ H_{e}=\sqrt{\left|S^{2}-E_{n}^{2}\right|} \end{array}\right.$$ (14)

#### 4.2.2. Evaluation steps

(1) Determine the factor set and the comment set. According to the evaluation index system, the factor set is determined as *U* = {*X*_1_, *X*_2_,……,*X*_*j*_}, evaluation set is *V* = {*V*_1_, *V*_2_, *V*_3_, *V*_4_, *V*_5_}. The interval is divided into [0,100], which corresponds to the domain [B_min_, B_max_].

(2) Determine the standard cloud. This paper uses the golden section idea to determine the standard cloud model for evaluation [49]. *B*_*min*_ and *B*_*max*_ are the minima and maximum boundaries respectively. *He* is a constant, which can be adjusted according to the ambiguity of the variable. The numerical characteristics of the cloud model for each evaluation interval can be calculated according to Table 9.

**Table 9:** Determine the standard cloud by golden section method.

Cloud	*Ex*	*En*	*He*
*E*_−2_	*B*_*min*_	*En*_1_/0.618	*He*_1_/0.618
*E*_−1_	*Ex*_0_−0.382(*B*_*min*_+*B*_*max*_)/2	0.382(*B*_*max*_−*B*_*min*_)/6	*He*_0_/0.618
*E*_0_	(*B*_*min*_+*B*_*max*_)/2	0.618*En*_1_	*He*_0_
*E*_1_	*Ex*_0_+0.382(*B*_*min*_+*B*_*max*_)/2	0.382(*B*_*max*_−*B*_*min*_)/6	*He*_0_/0.618
*E*_2_	*B*_*max*_	*En*_1_/0.618	*He*_1_/0.618

(3) Based on the public scores (It is hereby declared that this questionnaire survey was conducted in strict accordance with the guidelines and regulations, and was approved by the Medical Ethics Expert Committee of Shandong Provincial Health Commission, with the informed consent of all persons involved in the investigation.), using the fusion algorithm of cloud model [54], the single-factor evaluation cloud is integrated, and the comprehensive cloud digital characteristics of the implementation effect evaluation of public participation are obtained:

$$\left\{ \begin{array}{c} Ex=\sum_{j=1}^{n}E_{xj}w_{j}\\ En=\sqrt{\sum_{j=1}^{n}E_{nj}^{2}w_{j}}\\ He=\sum_{j=1}^{n}H_{ej}w_{j} \end{array}\right.$$ (15)

Then input *C*(*Ex*, *En*, *He*) into the forward cloud generator to generate a cloud map.

(4) Finally, the comprehensive cloud map obtained according to the actual data is compared with the standard cloud map, and the grade with the highest coincidence degree is the final evaluation grade of the implementation effect of public participation.

## 5. Case study

**Fig 4:**
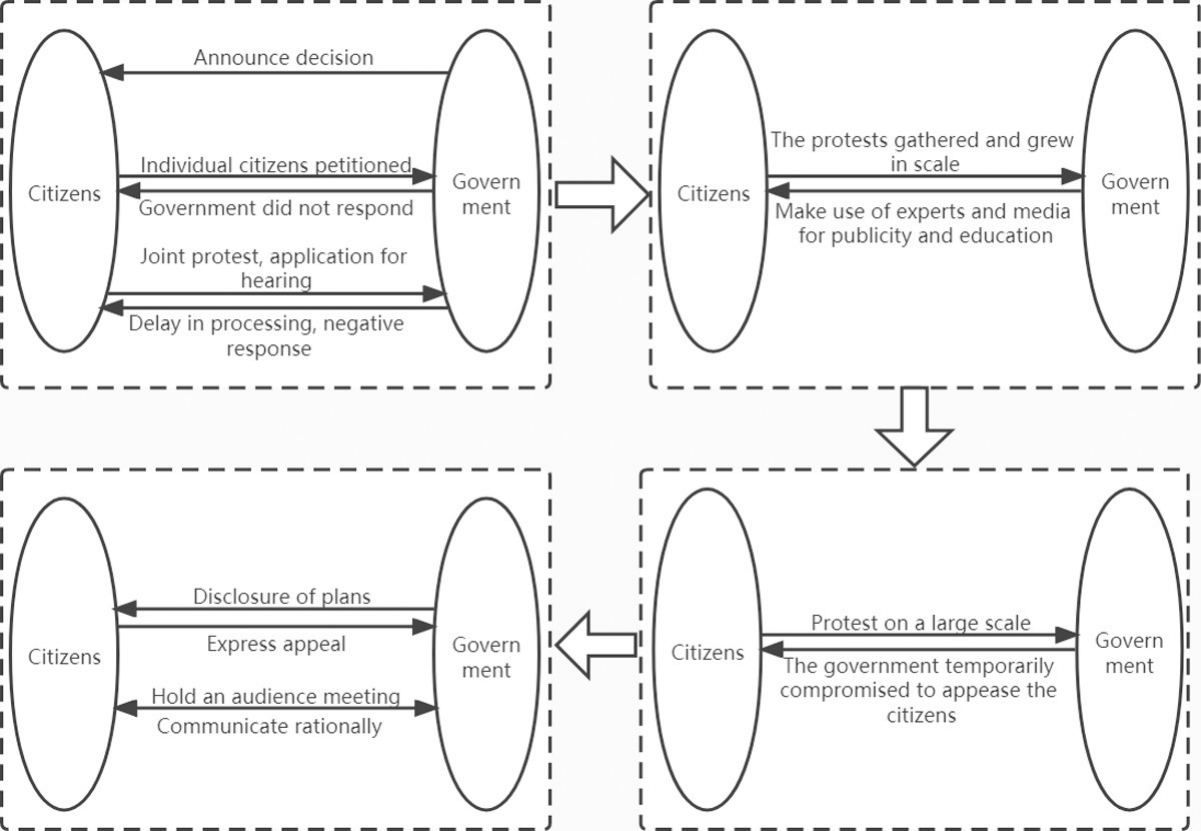
Interaction between the public and government in JiuFeng WTE plant project.

Our research area is located in Hangzhou, Zhejiang Province, China. With the increasing waste in Hangzhou and the construction of the Future Science and Technology City, the Planning Bureau has proposed building the Jiufeng WTE plant in the western part of the city. Jiu Feng WTE plant project is led by the Hangzhou Municipal Government and undertaken by Everbright International, Hangzhou Urban Construction Investment Group of Zhejiang Province, and Yuhang Urban Construction Group. The project covers an area of 34.43 acres with a total investment of approximately RMB 1.8 billion. The total designed scale is to handle 3,000 tons of domestic garbage per day, mainly responsible for the garbage in the downtown area of Hangzhou. After 18 months of construction, it was put into commercial operation on April 8, 2018, and can generate about 390 million kilowatt-hours of electricity per year. The construction of the Jiu Feng waste-to-energy plant has gone through twists and turns (Fig 4). Since the “Zhong Tai Incident” in 2014, this project has received great attention. Finally, the project was successfully built on the original site and put into operation smoothly, becoming a typical representative of NIMBY facility construction. Residents have experienced the typical construction of NIMBY facilities, constantly playing games with the relevant government departments. Therefore, this paper takes the project as a case to evaluate the implementation effect of public participation, which is a critical reference.

### 5.1. Combination weight based on DEMATEL and EWM

#### 5.1.1. Subjective weights

In this paper, 10 experts in related fields are invited to score independently according to the DEMATEL method, to determine the weights of indicators as accurately as possible, the respondents selected satisfy the following conditions: the experts have more than 5 years of practical experience in NIMBY facility projects; professors from well-known universities with more than 3 years of research on NIMBY facilities; officials in government departments who promote the construction of NIMBY facilities; senior management personnel responsible for the construction of NIMBY facilities in the enterprise.

Finally, 10 experts were selected as shown in Table 10. The index system was send to qualified experts to judge the importance of each factor, adopting 0–5 scoring method. If the factor *i* does not directly influence the factor *j*, then the corresponding score in the matrix was recorded as 0. Similarly, 1, 2, 3, 4, and 5 respectively very weak influence, weak influence, moderate influence, strong influence, and very strong influence respectively.

**Table 10:** Experts information.

No.	Work unit	Position	Working years
1	Tsinghua university	Professor	19 years
2	Shandong university	Professor	23 years
3	China Everbright International Limited	General manager	18 years
4	Environmental Impact Assessment Office of Tsinghua University	Engineer	25 years
5	Beijing Development and Reform Commission	Official	17 years
6	Shandong environmental protection consulting company	Professional consultant	21 years
7	Zhong Lun Law Firm	Lawyer	16 years
8	Municipal Administration Commission of Changping District of Beijing Municipality	Official	20 years
9	Southeast University	Professor	19 years
10	Hangzhou Ecological Environment Bureau	Official	22 years

Applying Eqs (1) and (2) to sort out the results of experts’ evaluation of the influence degree among indicators, and the normalized direct influence matrix is obtained as follows.


$$S=\left(\begin{array}{c} 0.000\;0.075\;0.064\;0.113\;0.000\;0.094\;0.064\;0.075\;0.075\;0.045\;0.113\;0.038\;0.038\;0.038\;0.091\;0.075\\ 0.019\;0.000\;0.038\;0.057\;0.038\;0.113\;0.057\;0.056\;0.056\;0.057\;0.075\;0.038\;0.038\;0.000\;0.113\;0.094\\ 0.026\;0.068\;0.000\;0.075\;0.045\;0.083\;0.079\;0.038\;0.060\;0.064\;0.079\;0.038\;0.038\;0.038\;0.094\;0.087\\ 0.038\;0.075\;0.000\;0.000\;0.019\;0.000\;0.057\;0.102\;0.038\;0.113\;0.106\;0.000\;0.000\;0.019\;0.113\;0.075\\ 0.008\;0.057\;0.038\;0.057\;0.000\;0.075\;0.042\;0.038\;0.000\;0.060\;0.072\;0.038\;0.023\;0.011\;0.079\;0.038\\ 0.038\;0.075\;0.057\;0.000\;0.038\;0.000\;0.072\;0.034\;0.034\;0.068\;0.113\;0.000\;0.038\;0.038\;0.128\;0.042\\ 0.026\;0.038\;0.045\;0.042\;0.038\;0.045\;0.000\;0.075\;0.064\;0.075\;0.038\;0.075\;0.057\;0.038\;0.038\;0.019\;\\ 0.057\;0.042\;0.053\;0.057\;0.000\;0.038\;0.038\;0.000\;0.075\;0.057\;0.057\;0.038\;0.038\;0.030\;0.087\;0.113\\ 0.038\;0.039\;0.075\;0.000\;0.038\;0.000\;0.057\;0.075\;0.000\;0.042\;0.075\;0.038\;0.038\;0.038\;0.109\;0.102\\ 0.030\;0.045\;0.038\;0.038\;0.038\;0.000\;0.045\;0.038\;0.094\;0.038\;0.057\;0.030\;0.023\;0.038\;0.057\;0.075\\ 0.000\;0.038\;0.064\;0.075\;0.049\;0.075\;0.075\;0.075\;0.038\;0.038\;0.000\;0.038\;0.038\;0.000\;0.000\;0.094\\ 0.038\;0.026\;0.068\;0.113\;0.042\;0.113\;0.068\;0.000\;0.038\;0.034\;0.113\;0.000\;0.075\;0.038\;0.094\;0.075\\ 0.038\;0.000\;0.049\;0.113\;0.000\;0.075\;0.053\;0.000\;0.038\;0.030\;0.075\;0.113\;0.000\;0.038\;0.083\;0.057\\ 0.008\;0.026\;0.068\;0.075\;0.000\;0.075\;0.038\;0.000\;0.019\;0.000\;0.075\;0.075\;0.075\;0.000\;0.064\;0.075\\ 0.008\;0.068\;0.038\;0.038\;0.075\;0.000\;0.075\;0.023\;0.000\;0.057\;0.000\;0.000\;0.000\;0.000\;0.001\;0.083\\ 0.038\;0.057\;0.068\;0.075\;0.000\;0.049\;0.094\;0.079\;0.064\;0.072\;0.060\;0.038\;0.075\;0.038\;0.098\;0.000 \end{array}\right)$$

Calculate the comprehensive impact matrix *T* by Eq (3).


$$T=\left(\begin{array}{c} 0.108\;0.268\;0.252\;0.325\;0.125\;0.278\;0.305\;0.273\;0.255\;0.277\;0.368\;0.176\;0.182\;0.136\;0.391\;0.356\\ 0.109\;0.166\;0.197\;0.235\;0.142\;0.262\;0.259\;0.220\;0.206\;0.251\;0.288\;0.151\;0.158\;0.085\;0.362\;0.323\\ 0.122\;0.241\;0.173\;0.269\;0.156\;0.250\;0.294\;0.216\;0.221\;0.272\;0.309\;0.164\;0.169\;0.127\;0.364\;0.334\\ 0.115\;0.215\;0.143\;0.162\;0.110\;0.137\;0.232\;0.246\;0.176\;0.280\;0.282\;0.104\;0.108\;0.090\;0.324\;0.284\\ 0.074\;0.178\;0.155\;0.192\;0.080\;0.192\;0.193\;0.160\;0.115\;0.206\;0.231\;0.122\;0.113\;0.073\;0.265\;0.213\\ 0.113\;0.218\;0.199\;0.169\;0.133\;0.146\;0.251\;0.180\;0.169\;0.237\;0.295\;0.110\;0.146\;0.109\;0.342\;0.253\\ 0.105\;0.175\;0.184\;0.203\;0.125\;0.185\;0.174\;0.210\;0.194\;0.237\;0.230\;0.177\;0.162\;0.111\;0.260\;0.226\\ 0.140\;0.195\;0.205\;0.232\;0.098\;0.187\;0.232\;0.159\;0.218\;0.238\;0.261\;0.149\;0.155\;0.110\;0.325\;0.331\\ 0.118\;0.186\;0.221\;0.177\;0.131\;0.152\;0.242\;0.221\;0.141\;0.216\;0.266\;0.149\;0.152\;0.113\;0.333\;0.313\\ 0.103\;0.176\;0.171\;0.190\;0.121\;0.132\;0.210\;0.175\;0.217\;0.194\;0.232\;0.129\;0.125\;0.106\;0.263\;0.268\\ 0.082\;0.178\;0.199\;0.231\;0.133\;0.210\;0.247\;0.217\;0.172\;0.209\;0.193\;0.140\;0.145\;0.076\;0.227\;0.290\\ 0.134\;0.208\;0.240\;0.312\;0.155\;0.284\;0.291\;0.186\;0.201\;0.250\;0.350\;0.131\;0.206\;0.129\;0.370\;0.328\\ 0.119\;0.153\;0.195\;0.281\;0.098\;0.220\;0.241\;0.153\;0.175\;0.210\;0.278\;0.214\;0.115\;0.115\;0.314\;0.270\\ 0.082\;0.160\;0.197\;0.230\;0.087\;0.209\;0.208\;0.135\;0.142\;0.161\;0.256\;0.171\;0.176\;0.070\;0.273\;0.264\\ 0.058\;0.158\;0.124\;0.141\;0.130\;0.093\;0.185\;0.119\;0.090\;0.169\;0.123\;0.069\;0.071\;0.048\;0.146\;0.208\\ 0.134\;0.228\;0.236\;0.272\;0.112\;0.217\;0.306\;0.252\;0.228\;0.277\;0.291\;0.167\;0.203\;0.128\;0.366\;0.256 \end{array}\right)$$

**Fig 5:**
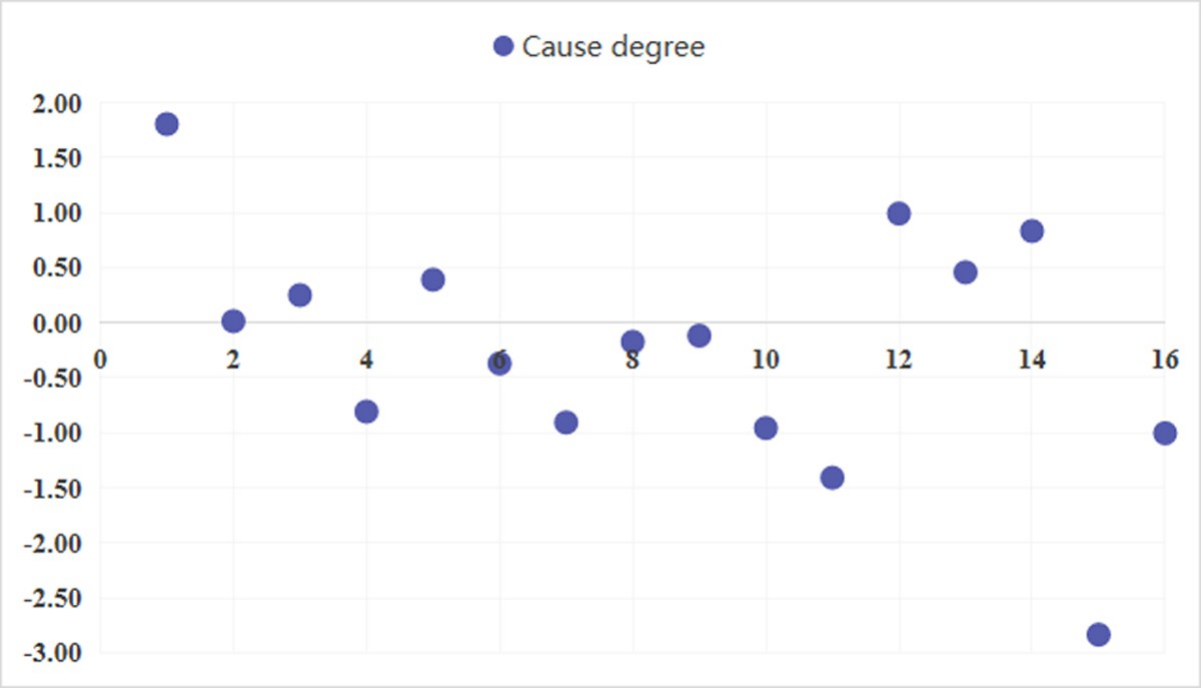
Scatter plot of the cause degree.

According to the comprehensive impact matrix *T*, calculate the effect degree *E*_*i*_, affected degree *P*_*i*_, center degree *M*_*i*_ and cause degree *C*_*i*_, and determine the subjective weight w′_k_ of each index. To provide a clear distinction between each influencing factor, the cause degree “*E*−*P*” in the scatter plot (as depicted in Fig 5), is drawn according to the value listed in Table 11.

**Table 11:** Cause and effect values.

Index	Effect degree	Affected degree	Center degree	Cause degree	Index weight
C_1_	3.3280	1.5234	4.8514	1.8046	0.0594
C_2_	2.7301	2.7165	5.4466	0.0136	0.0625
C_3_	2.9815	2.7308	5.7122	0.2507	0.0656
C_4_	2.4004	3.2084	5.6088	-0.8079	0.0651
C_5_	2.0840	1.6933	3.7774	0.3907	0.0436
C_6_	2.4744	2.8449	5.3192	-0.3705	0.0612
C_7_	2.4717	3.3782	5.8499	-0.9065	0.0680
C_8_	2.5777	2.7512	5.3290	-0.1735	0.0612
C_9_	2.4843	2.6021	5.0864	-0.1177	0.0584
C_10_	2.2809	3.2382	5.5191	-0.9573	0.0643
C_11_	2.4300	3.8382	6.2682	-1.4082	0.0738
C_12_	3.0781	2.0865	5.1646	0.9916	0.0604
C_13_	2.5681	2.1102	4.6782	0.4579	0.0540
C_14_	2.2848	1.4522	3.7369	0.8326	0.0440
C_15_	1.5794	4.4138	5.9931	-2.8344	0.0761
C_16_	3.0510	4.0548	7.1057	-1.0038	0.0824

As exhibited in Table 11, 7 risk factors are identified with positive “*E*−*P*” value, including the soundness of relevant laws and regulations (C_1_ = 1.805), project information openness and transparency (C_2_ = 0.014), the government’s attitude towards public participation (C_3_ = 0.251), the level of attention from the news media (C_5_ = 0.391), involvement of NGOs (C_12_ = 0.992), the role of relevant experts (C_13_ = 0.458), and the objective attitude of EIA agencies (C_14_ = 0.833). This suggests that the influence of these factors on other factors are more significant than the influence that others exert on themselves. In addition, 9 risk factors have negative “*E*−*P*” values. These factors consist of mechanism for receiving public feedback (C_4_ = -0.808), the convenience of project information access (C_6_ = -0.371), public awareness of participation (C_7_ = -0.907), interactivity of public participation (C_8_ = -0.174), representation of the main body of public participation (C_9_ = -0.118), continuity of public participation (C_10_ = -0.957), transparency in the participation process (C_11_ = -1.408), cost consumption in public participation (C_15_ = -2.834), and the influence of public opinion on decision-making (C_16_ = -1.0038). On the contrary, the result is reflective of the fact that the influence of these risk factors on other factors is less than the influence that others impose on themselves. As summarized in Table 6, almost all of the basis of participation and external support factors with positive “*E*−*P*” value are in the cause group, whereas participation process and cost-effectiveness factors with negative value are in the effect group. Therefore, it means that the basis of participation and external support factors significantly affect other factors, while the participation process and cost-effectiveness factors are prominently influenced by other factors, which is in line with real conditions.

The center degree “*E*+*P*” represents the importance of each factor in the overall analysis structure. The center degree of the 16 factors rank from the largest to the smallest as follows: C_16_, C_11_, C_15_, C_7_, C_3_, C_4_, C_10_, C_2_, C_8_, C_6_, C_12_, C_9_, C_1_, C_13_, C_5_, C_14_. In the view of the “*E*+*P*” value, seven critical factors from the cause group can be attained in order: the government’s attitude towards public participation (C_3_)>project information openness and transparency (C_2_)>involvement of NGOs (C_12_)> the soundness of relevant laws and regulations (C_1_)>the role of relevant experts (C_13_)>the level of attention from the news media (C_5_)>and the objective attitude of EIA agencies (C_14_). Finally, the weight of each factor is obtained by combining the center and cause degree.

#### 5.1.2. Objective weights

After the standardizing the original data, the entropy value of each index can be obtained as 0.9979, 0.9977, 0.9979, 0.9989, 0.9978, 0.9979, 0.9977, 0.9978, 0.9979, 0.9978, 0.9978, 0.9979, 0.9976, 0.9980, 0.9979, 0.9987, 0.9977, 0.9976 respectively through Eq (5). Finally, the objective weights of indicators can be obtained as $w_{k}^{\prime \prime }$ = (0.0649, 0.0666, 0.0646, 0.0660, 0.0334, 0.0667, 0.0636, 0.0669, 0.0652, 0.0637, 0.0711, 0.0615, 0.0630, 0.0398, 0.0702, 0.0726) by employing Eq (6).

#### 5.1.3 Combination weight

According to the combination weighting principle of game theory, the linear combination coefficients $\lambda_{1}^{*}=0.427$ and $\lambda_{2}^{*}=0.573$ are calculated by Eqs (9) and (10), and the final combination weight of each index w* = (0.0626, 0.0649, 0.0651, 0.0656, 0.0377, 0.0644, 0.0655, 0.0645, 0.0623, 0.0640, 0.0722, 0.0610, 0.0592, 0.0416, 0.0727, 0.0768) is obtained.

### 5.2. Apply cloud model for evaluation

This research uses the established evaluation index system to evaluate the implementation effect of public participation in the Jiu Feng WTE plant project. In the interval [0,100], the comment set is divided into V = {very poor, poor, average, good, very good} based on the five-point Likert scale, and the corresponding (*Ex*, *En*, *He*) is calculated according to the golden section method given in Table 10. The results are shown in Table 12.

**Table 12:** Cloud model division evaluation cloud.

Evaluation degree	Grade range	Cloud model parameters
Very poor	[0, 20)	(0.0, 10.30, 1.31)
Poor	[20, 40)	(30.9, 6.37, 0.81)
General	[40, 60)	(50.0, 3.94, 0.50)
Good	[60, 85)	(69.1, 6.37, 0.81)
Very good	[85,100]	(100.0, 10.30, 1.31)

**Fig 6:**
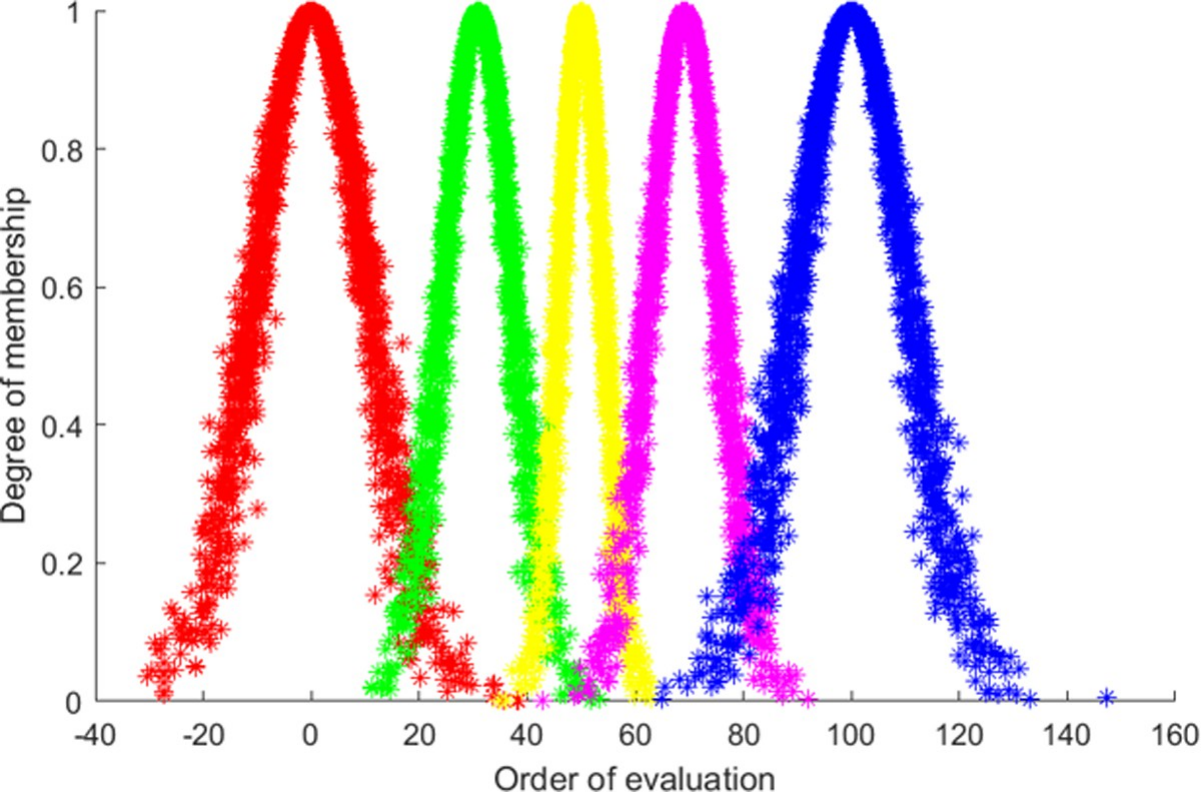
The standard cloud map.

According to the cloud model digital characteristic parameter of each evaluation level, the standard cloud map corresponding to each evaluation grade is generated by MATLAB (as shown in Fig 4). From left to right, the corresponding evaluation levels are successive “very poor”, “poor”, “general”, “good” and “very good”. This is shown in Fig 6.

In order to more truly reflect the implementation effect of public participation, here we conducted surveys and visits to residents and related personnel near Hangzhou Jiu Feng WTE plant. The public and related personnel scored 16 evaluation indexes in S3 File according to the divided grade interval. In terms of the scoring situation, the numerical characteristics of the index are calculated by using the reverse generator of cloud model and formulas (11)-(14), as shown in Table 13.

**Table 13:** Digital characteristics of the cloud model.

Indicators	(*Ex*, *En*, *He*)	Weight
C_1_	(61.043, 3.312, 0.339)	0.0626
C_2_	(60.675, 3.578, 0.410)	0.0649
C_3_	(58.493, 3.456, 0.799)	0.0651
C_4_	(59.418, 2.297, 0.687)	0.0656
C_5_	(60.238, 2.052, 0.669)	0.0377
C_6_	(59.433, 2.109, 0.445)	0.0644
C_7_	(63.008, 2.184, 1.178)	0.0655
C_8_	(54.663, 2.930, 0.794)	0.0645
C_9_	(56.323, 3.324, 0.913)	0.0623
C_10_	(56.100, 3.635, 0.451)	0.0640
C_11_	(58.745, 2.939, 0.697)	0.0722
C_12_	(50.785, 2.864, 0.733)	0.0610
C_13_	(61.625, 3.039, 0.485)	0.0592
C_14_	(60.578, 2.178, 0.702)	0.0416
C_15_	(58.500, 2.507, 0.847)	0.0727
C_16_	(54.063, 2.804, 0.319)	0.0768

**Fig 7:**
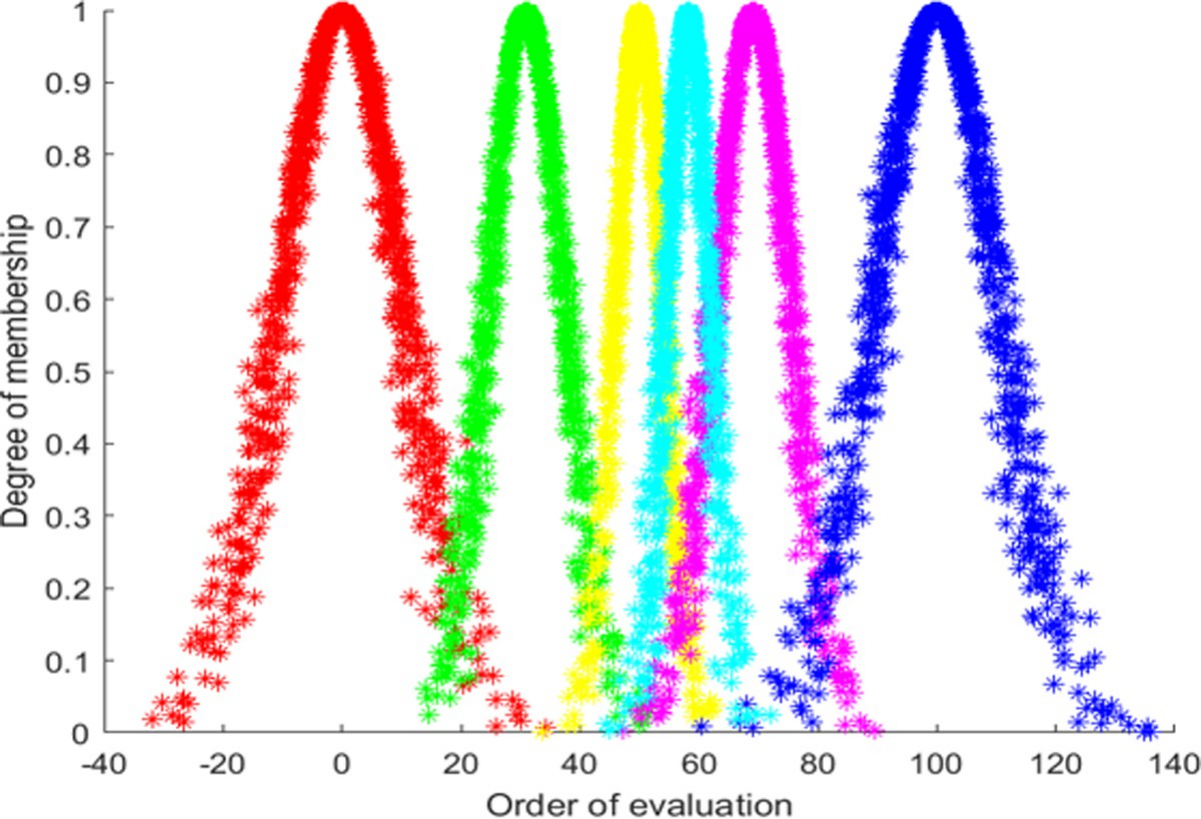
Comprehensive evaluation cloud map.

Employing Eq (15) to integrate the digital parameter characteristics of the index, the digital parameter characteristics of the cloud model for comprehensive evaluation is calculated as *C*(59.223,2.900,0.652). And input this parameter into the forward cloud generator, then the comprehensive evaluation cloud map is drawn by using MATLAB, as shown in Fig 7. From the cloud map presented by the evaluation results, it can be seen that the implementation effect of public participation in the project is between “general” and “good”, and there is still a large space for improvement, which is consistent with the actual situation of Jiu Feng WTE plant construction, and verifies that the evaluation model constructed in this paper has good practicability and effectiveness. To further identify the problems, we analyzed the secondary indicators.

In the category of the basis of participation, the *Ex* of the soundness of relevant laws, the government’s attitude towards the public participation, and convenience of project information access are larger, which indicates that due to the perfection of the relevant legal system and the importance of the construction unit, the information disclosure work is better. At the same time, with the development of information technology and the popularization of the Internet, it is more convenient for the public to obtain project-related information. In addition, the *Ex* and *He* of project information openness and transparency, mechanism for receiving public feedback and the level of attention from the news media is also greater, indicating that there is disagreement on these indicators and the work in these areas needs to be strengthened, so the disclosure of information should be targeted and useful to the public, and should not be partially disclosed and avoid the key issues. Relevant government departments can also make full use of the Internet platform to build scientific and convenient public participation platforms, such as opening dedicated participation channels on Weibo and WeChat platforms. On the one hand, it is convenient for the government to announce project-related information and promote more public willingness to participate, so as to fully collect public suggestions and realize two-way communication. On the other hand, the public is also able to follow and fully supervise the government’s work and project decision-making process through the platform, thus realizing the convenience of participation channels. The news media should play a greater role in project decision-making, provide the public with timely information about the project, play a good monitoring role, and at the same time do a good job of publicity to help raise public awareness of participation.

In terms of participation process, *Ex* of public participation awareness and transparency of public participation is greater, but *He* is larger, which indicates that public participation awareness has awakened with the progress of society, but public participation awareness should be further improved to strive for reasonable rights and interests more actively. In addition, other indicators with smaller *Ex* and larger *En* or *He* have big room for improvement, which is mainly due to the fact that the current public participation is in the primary stage and the public only participates symbolically. Therefore, when selecting public representatives, the coverage of different groups should be fully considered, and the government should pay attention to forming a positive interaction with the public.

In terms of external support, the *Ex* for involvement of NGOs is low and currently plays a weak role, with very significant room for improvement. Compared to other organizations, environmental NGOs are third-party organizations that are decoupled from government interests and not directly involved with public interests, and their position is more impartial. Introducing them into the project decision-making stage can reduce public concerns. At the same time, compared with the general public, NGOs have stronger expertise and ability. On the one hand, they can convey the government’s wishes to the public, on the other hand, they can help the public integrate their internal opinions and convey them to the government, serving as a two-way communication bridge between the government and the public, thus better guiding public participation, avoiding deviations in the public’s individual behavioral intentions, and promoting the healthy development of public participation. However, due to the difficulties of insufficient funding, social resources, and the lack of actual administrative authority and legal protection, Chinese environmental NGOs are currently small in scale and struggle to play a large role. Therefore, the government should support NGOs from the object environment and give them more autonomy so that they can carry out their activities comfortably within the lawful scope and form a culture of pluralistic co-governance between the government and NGOs. In addition, the government should strengthen the capacity building of the main body of NGOs and guide them to enhance participation capacity by participating in dispute coordination meetings and providing advice and environmental knowledge to the public, so as to alleviate the public’s over-exaggerated environmental anxiety, improve their credibility in the public’s mind.

In terms of cost-effectiveness, the *Ex* of the public’s influence on decision-making is small, indicating that the public believes that their opinions have not been fully adopted and did not have sufficient influence on the decision-making. In addition, the *Ex* of cost consumption in public participation is large, and the *En* and *He* are relatively small, which indicates that the economic and time costs invested by the public in the participation process are generally reasonable, and the participation process does not cost too much money or take up too much time and energy of the participants.

## 6. Discussion

### 6.1 Sensitivity analysis

In order to verify the robustness of the evaluation method, a sensitivity analysis is conducted. In this paper, the first-level indicators are made to change ±10%, ±20% and ±30%, respectively, to simulate the change of decision makers’ preference for different indicators under disparate situations, and compare the impact of these changes on the evaluation result cloud, red, green and blue respectively represent the cloud of ±10%, ±20%, and ±30% change of indicators.

**Fig 8:**
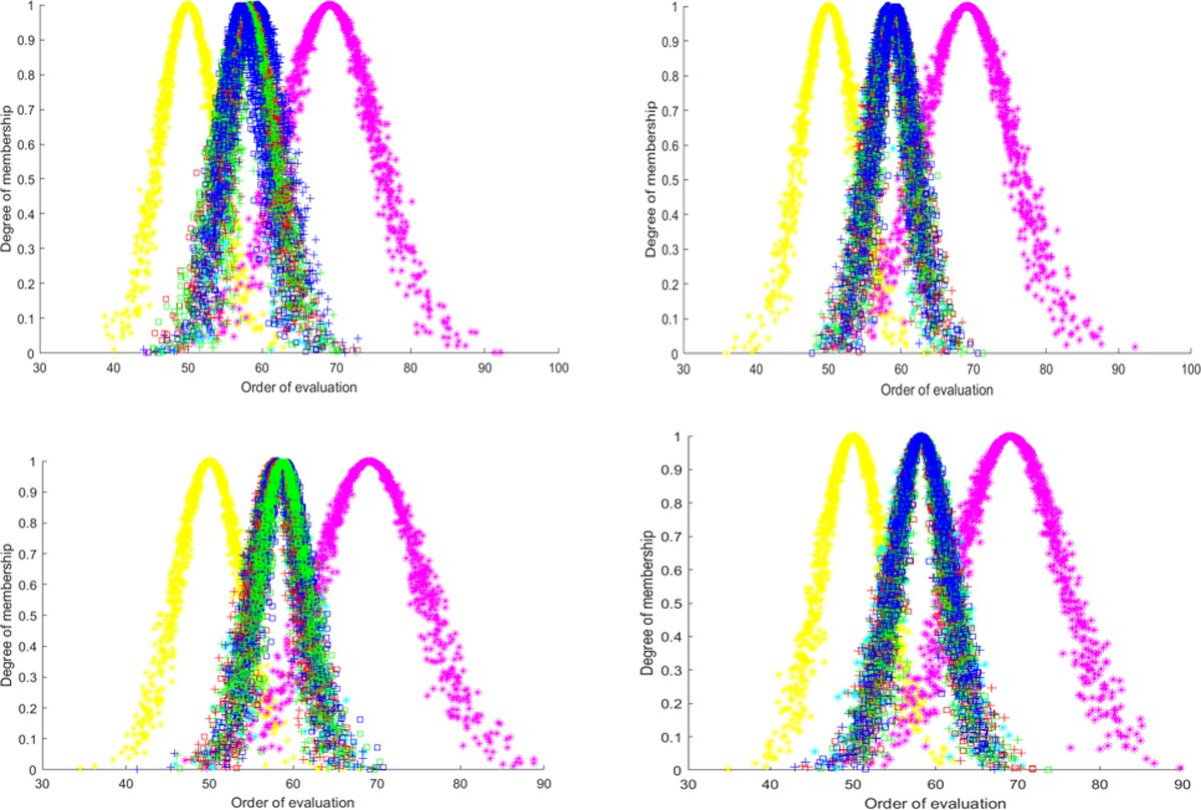
Cloud map of sensitivity analysis.

In Fig 8, the evaluation result clouds all change slightly during the sensitivity analysis, but the closeness is still between average and good, which proves that the evaluation model proposed has good stability and scientific validity. Combining the comparison between the graphical and digital features of these evaluation clouds, it can be seen that although the evaluation results are stable under each change, the change in index C1 has a more obvious impact on the results, which is due to the fact that the component containing the indicator is more important. Meanwhile, the evaluation results all change with the four indicators, and the cloud thickness and dispersion are larger for a 30% increase in weight, which is caused by the larger value of the resultant cloud.

### 6.2 Comparative analysis

To further illustrate the effectiveness and practicability of this method, this paper compares it with the best-worst method, one of the latest multi-criteria decision-making methods. The BWM was proposed by Rezaei J in 2015 [55], in which experts (decision-makers) select the best (most important) and worst (least significant) criteria based on experience and actual engineering needs, and then compare the best (worst) criteria with each of the remaining indicators in turn. After comparison, each indicator will form an integer value from 1 to 9, reflecting the relative degree of superiority or inferiority. Finally, the BWM solution can be transformed into a mathematical planning problem, and the results can be calculated using Lingo software. Kumar P et.al [56] explored the challenges in sustainable supply chain of electric vehicle batteries and determined the priority of these challenges by using the BWM method. Kusi-Sarpong S et.al [57] adopted a multi-criteria decision-making method to evaluate and select sustainable suppliers, using weights determined by BWM to rank the suppliers. At the same time, the BWM has also been applied in many cases, such as choosing the right place to set up temporary hospitals for Covid-19 patients [58], assessing renewable energy potential [59], evaluating the risk of PPP waste-to-energy incineration plant projects [60], and assessing energy security performances [61].

**Fig 9:**
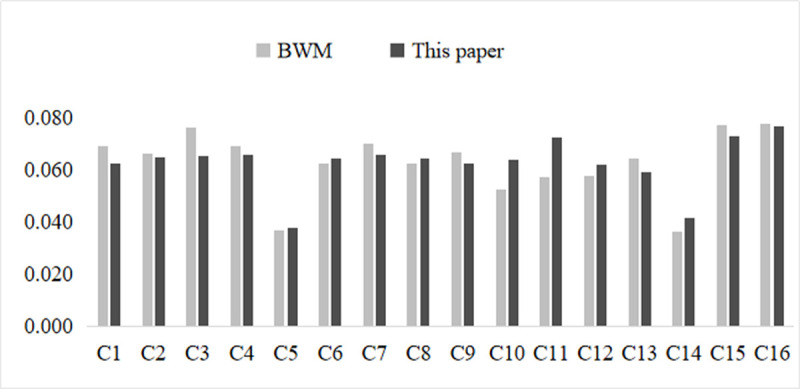
Weights comparison.

The weights obtained using BWM are shown in Table 14, and further comparison Fig 9 is drawn, from which it can be seen that the weights have some differences but are basically the same, indicating the effectiveness of the proposed method. Although BWM can obtain roughly the same weights, they cannot deal with the interrelationship between criteria and cannot overcome the human factor of subjective assignment. Compared with the above-mentioned method, our proposed method can take into account the influence relationship between indicators, which can incorporate the theories and experiences of senior experts, while striving to reduce the subjective arbitrariness of the assignment and make the evaluation results more realistic. Therefore, the proposed method has strong generality and flexibility for judging the implementation effect of public participation in the decision-making of NIMBY facilities.

**Table 14:** Weights obtained through BWM.

First-level factors	Second-level factors	Weights of first-level factors	Weights of second-level factors	Weights of factors
B_1_	C_1_	0.3789	0.1826	0.0692
	C_2_		0.1741	0.0660
	C_3_		0.2005	0.0760
	C_4_		0.1817	0.0688
	C_5_		0.0963	0.0365
	C_6_		0.1648	0.0624
B_2_	C_7_	0.3080	0.2272	0.0700
	C_8_		0.2025	0.0624
	C_9_		0.1740	0.0536
	C_10_		0.1694	0.0522
	C_11_		0.2269	0.0699
B_3_	C_12_	0.1584	0.3640	0.0577
	C_13_		0.4073	0.0645
	C_14_		0.2287	0.0362
B_4_	C_15_	0.1547	0.4984	0.0771
	C_16_		0.5016	0.0776

## 7. Conclusion

The evaluation framework constructed in this paper has important implications for research on the implementation effect of public participation of NIMBY facilities, which can identify the barriers affecting the implementation effect of public participation and provide policymakers with references to optimize the public participation system. Based on the characteristics of NIMBY facilities, this research constructs an evaluation index system for the implementation effect of public participation through literature review and questionnaires. In order to make the weights more reasonable, DEMATEL and EWM are provided to get the combined weights of factors. To a certain extent, it can overcome the disadvantages of subjective weight. Furthermore, the method can analyze the causal relationship among the factors influencing the implementation effect. It can be found that the basis of participation and external support factors significantly affect other factors, while the participation process and cost-effectiveness factors are prominently influenced by other elements. Finally, a case is selected to demonstrate the effectiveness of the new model. It not only considers the uncertainty of evaluation factors but also takes into account the fuzziness of evaluation criteria, which can obtain more intuitive, accurate, and objective results. We provide a quantitative evaluation method to promote public participation in the decision-making of NIMBY facilities. This paper found that the awareness of public participation has been awakened in polluting NIMBY facilities. However, the participation process lacks continuity and the interaction with the government is insufficient. To solve this problem, different groups should be fully considered when choosing public representatives. At the same time, relevant government departments should make full use of the Internet platform, build a scientific and convenient platform for public participation, and open special participation channels. In addition, with government support, there are reasons to believe that NGOs will play a more significant role.

This research also has several limitations and shortcomings. Due to insufficient public participation experience in NIMBY facilities, it is impossible to improve the evaluation index system of the implementation effect of public participation. Secondly, the collected data mainly come from subjective scoring. Although using the cloud model can reduce the subjectivity of scoring results to a certain extent, the lack of objective indicators may still cause the evaluation results to be incomplete. In the following research phase, the identification method of the factors influencing the implementation effect of public participation can be innovated. Some objective and quantitative evaluation indicators, such as the number of hearings held and the expenditure related to the public involvement, can be added to the evaluation index system to make it more scientific, comprehensive, and better reflect the implementation effect of public participation.

## Supporting information

S1 Data The questionnaire 1, 2 data, DEMATEL, and BWM data. (XLSX)

S1 File Preliminary statistics of evaluation indicators. (DOCX)

S2 File Questionnaire on influencing factors of implementation effect of public participation in decision-making of polluting NIMBY facilities. (DOCX)

S3 File Questionnaire on implementation effect of Public participation of Hangzhou Jiufeng waste-to-energy plant. (DOCX)
