# Supplementary material for: Evaluation on the implementation effect of public participation in the decision-making of NIMBY facilities
Source: PLoS One. 2022 Feb 18;17(2):e0263842. doi: 10.1371/journal.pone.0263842 (PMC8856540; doi:10.1371/journal.pone.0263842)
Supplement: S1 File — (DOCX) [file pone.0263842.s002.docx]

**S2. Preliminary statistics of evaluation indicators.**

| **No.** | **Influence factors** |
| --- | --- |
| 1 | Project information openness and transparency |
| 2 | The government's attitude towards public participation |
| 3 | The soundness of relevant laws and regulations |
| 4 | Social and economic status of the public |
| 5 | A mechanism for receiving public feedback |
| 6 | The level of attention from the news media |
| 7 | Convenience of project information access |
| 8 | Public's understanding of NIMBY facilities |
| 9 | Public awareness of participation |
| 10 | The interactivity of public participation |
| 11 | Representation of the main body of public participation |
| 12 | Involvement of NGOs |
| 13 | Transparency in the participation process |
| 14 | Continuity of public participation |
| 15 | The role of relevant experts |
| 16 | The influence of public opinion on decision-making |
| 17 | Cost consumption in public participation |
| 18 | The objective attitude of EIA agencies |
