# Supplementary material for: Evaluation on the implementation effect of public participation in the decision-making of NIMBY facilities
Source: PLoS One. 2022 Feb 18;17(2):e0263842. doi: 10.1371/journal.pone.0263842 (PMC8856540; doi:10.1371/journal.pone.0263842)
Supplement: S2 File — (DOCX) [file pone.0263842.s003.docx]

**S3. Questionnaire on influencing factors of implementation effect of public participation in decision-making of polluting NIMBY facilities.**

Dear Sir/Madam,

Please judge the importance of each factor based on your own experience. If you think there are other important factors, you can add them at the end. The time and energy you contributed to this survey will have a very important effect on us. Thank you very much for your cooperation.

All the questions in this questionnaire will not involve your work secrets, and your basic information will be kept strictly confidential for academic research purposes only.

Gender: Male female

Age: 20-29 years 30-39 years 40-49 years ≥50 years

Work experience: 0-5 years 6-10 years ≥11 years

Educational background: High school and below Associate college Bachelor’s degree Master's degree and above

| Please mark "√" in the corresponding space | | | | | | | |
| --- | --- | --- | --- | --- | --- | --- | --- |
| N0. | Influencing factors | Explain | Very  unimportant | Unimportant | General | More important | Very important |
| 1 | Project information openness and transparency | Whether all information relating to the construction of the NIMBY facility is disclosed in a timely manner |  |  |  |  |  |
| 2 | The government's attitude towards public participation | Whether the government organizes professional training or consultation meetings for the public and whether it has a active attitude towards public participation |  |  |  |  |  |
| 3 | The soundness of relevant laws and regulations | The adequacy of laws and regulations that specifically address public participation in NIMBY facilities |  |  |  |  |  |
| 4 | A mechanism for receiving public feedback | The ability of the government to accept and respond to public opinion |  |  |  |  |  |
| 5 | The level of attention from the news media | Whether the media is monitoring the reporting of critical events and ensuring the authenticity of the reporting |  |  |  |  |  |
| 6 | Convenience of project information access | Whether the information disclosed by the NIMBY facility construction enterprise and the government is easy to obtain |  |  |  |  |  |
| 7 | Public awareness of participation | The degree of public awareness and concern about NIMBY facilities, and whether the public has the intention to participate in the construction of NIMBY facilities |  |  |  |  |  |
| 8 | The interactivity of public participation | Whether the public and the government can carry out information interaction |  |  |  |  |  |
| 9 | Representation of the main body of public participation | The fact that the public participants are representative enough to express the opinions of the public |  |  |  |  |  |
| 10 | Involvement of NGOs | The ability of environmental NGOs to act as a bridge between the government and the public while remaining neutral |  |  |  |  |  |
| 11 | Transparency in the participation process | Whether the process of public participation is open and transparent |  |  |  |  |  |
| 12 | Continuity of public participation | Whether the public has time to continue to participate in the decision-making of NIMBY facilities |  |  |  |  |  |
| 13 | The role of relevant experts | The objectivity and impartiality of experts involved in the decision-making of NIMBY facilities |  |  |  |  |  |
| 14 | The influence of public opinion on decision-making | Whether public opinion is taken into account in the final decision |  |  |  |  |  |
| 15 | Cost consumption in public participation | The cost of time and money for the public to get involved |  |  |  |  |  |
| 16 | The objective attitude of EIA agencies | The neutrality of the environmental assessment agency during the review process |  |  |  |  |  |
