# Supplementary material for: Evaluation on the implementation effect of public participation in the decision-making of NIMBY facilities
Source: PLoS One. 2022 Feb 18;17(2):e0263842. doi: 10.1371/journal.pone.0263842 (PMC8856540; doi:10.1371/journal.pone.0263842)
Supplement: S3 File — (DOCX) [file pone.0263842.s004.docx]

**S4. Questionnaire on implementation effect of Public participation of Hangzhou Jiufeng waste-to-energy plant.**

Dear Sir/Madam,

Thank you for your support and cooperation in this questionnaire survey in your busy schedule! The research team is conducting an investigation and analysis on the evaluation of the implementation effect of public participation in decision-making of polluting NIMBY facilities.Through this survey, I hope to know your real thoughts on the implementation effect of public participation of Jiufeng waste-to-energy plant project.

All the questions in this questionnaire will not involve your work secrets, and your basic information will be kept strictly confidential for academic research purposes only.

Gender: Male female

Age: 20-29 years 30-39 years 40-49 years ≥50 years

Work experience: 0-5 years 6-10 years ≥11years

Educational background: High school and below Associate college Bachelor’s degree Master's degree and above

Please write your grade in ( )

1.Do you think the laws and regulations related to NIMBY facilities are sound? ( )

A.Very unsound(0-19) B.Unsound(20-39) C.General(40-59) D.More sound(60-84) E.Very sound(85-100)

2.Do you think the government has disclosed all information related to the construction of NIMBY facilities in a timely manner?

A.Very late(0-19) B.Not timely(20-39) C.General(40-59) D.Timely(60-84) E.Very timely(85-100)

3.Do you think the government has a positive attitude towards public participation? ( )

A.Very inactive(0-19) B.Not active(20-39) C.General active(40-59) D.More active(60-84) E.Very active(85-100)

4.What do you think of the government's mechanism for accepting public feedback in this project? ( )

A.Very imperfect(0-19) B.Imperfect(20-39) C.General(40-59) D.Relatively perfect(60-84) E.Very perfect(85-100)

5.Do you think the news media have played a role in information disclosure and news supervision during the construction of the project? ( )

A.Very ineffective(0-19) B.No effect(20-39) C.General effect(40-59) D.Relatively large effect(60-84) E.Very large effect(85-100)

6.Do you think it is convenient to obtain project related information during the construction of the project? ( )

A.Very inconvenient(0-19) B.Inconvenient(20-39) C.General(40-59) D.Quite convenient(60-84) E.Very convenient(85-100)

7.Do you think the public's awareness of participation is positive during the construction of the project? ( )

A.Very inactive(0-19) B.Not active(20-39) C.General active(40-59) D.More active(60-84) E.Very active(85-100)

8.Do you think there is interaction between the public and the government? ( )

A.No interaction(0-19) B.Slightly interaction(20-39) C.Interaction(40-59) D.More interaction(60-84) E.A lot of interaction(85-100)

9.Do you think the main body of public participation is sufficiently representative during the construction of the project? ( )

A.Very unrepresentative(0-19) B.Unrepresentative(20-39) C.General representativeness(40-59) D.Strong representative(60-84) E.Very strong representative(85-100)

10.Do you think public participation covers the whole process of the project? ( )

A.No coverage(0-19) B.Less coverage(20-39) C.Partial coverage(40-59) D.Basic coverage(60-84) E.Full coverage(85-100)

11.Do you think the public participation process is transparent during the construction of the project? ( )

A.Very opaque(0-19) B.Opaque(20-39) C.General transparent(40-59) D.More transparent(60-84) E.Very transparent(85-100)

12.Do you think non-governmental organizations (such as environmental protection organizations) have played a role in the construction of the project? ( )

A.Very ineffective(0-19) B.No effect(20-39) C.General effect(40-59) D.Relatively large effect(60-84) E.Very large effect(85-100)

13.Do you think experts in relevant fields (such as environment and economy) have played a role in the construction of the project? ( )

A.Very ineffective(0-19) B.No effect(20-39) C.General effect(40-59) D.Relatively large effect(60-84) E.Very large effect(85-100)

14.Do you think the attitude of the EIA organization is objective during the construction of the project? ( )

A.Very not objective(0-19) B.A little not objective(20-39) C.Objective(40-59) D.More objective(60-84) E.Very objective(85-100)

15.Do you think the time, money and other costs of public participation in the construction of the project are reasonable? ( )

A.Too much, very unreasonable(0-19) B.Unreasonable(20-39) C.Reasonable(40-59) D.More reasonable(60-84) E.Very reasonable(85-100)

16.Do you think the public's opinions have affected the final decision of the project during the construction of the project? ( )

A.No impact(0-19) B.Small impact(20-39) C.Certain impact(40-59) D.Large impact(60-84) E.Very large impact(85-100)
